# Identification of Novel Regulatory Cholesterol Metabolite, 5-Cholesten, 3β,25-Diol, Disulfate

**DOI:** 10.1371/journal.pone.0103621

**Published:** 2014-07-29

**Authors:** Shunlin Ren, Jin Koung Kim, Genta Kakiyama, Daniel Rodriguez-Agudo, William M. Pandak, Hae-Ki Min, Yanxia Ning

**Affiliations:** Department of Medicine, Veterans Affairs McGuire Medical Center/Department of Medicine, Virginia Commonwealth University, Richmond, Virginia, United States of America; Boston University School of Medicine, United States of America

## Abstract

Oxysterol sulfation plays an important role in regulation of lipid metabolism and inflammatory responses. In the present study, we report the discovery of a novel regulatory sulfated oxysterol in nuclei of primary rat hepatocytes after overexpression of the gene encoding mitochondrial cholesterol delivery protein (StarD1). Forty-eight hours after infection of the hepatocytes with recombinant StarD1 adenovirus, a water-soluble oxysterol product was isolated and purified by chemical extraction and reverse-phase HPLC. Tandem mass spectrometry analysis identified the oxysterol as 5-cholesten-3β, 25-diol, disulfate (25HCDS), and confirmed the structure by comparing with a chemically synthesized compound. Administration of 25HCDS to human THP-1-derived macrophages or HepG2 cells significantly inhibited cholesterol synthesis and markedly decreased lipid levels in vivo in NAFLD mouse models. RT-PCR showed that 25HCDS significantly decreased SREBP-1/2 activities by suppressing expression of their responding genes, including ACC, FAS, and HMG-CoA reductase. Analysis of lipid profiles in the liver tissues showed that administration of 25HCDS significantly decreased cholesterol, free fatty acids, and triglycerides by 30, 25, and 20%, respectively. The results suggest that 25HCDS inhibits lipid biosynthesis via blocking SREBP signaling. We conclude that 25HCDS is a potent regulator of lipid metabolism and propose its biosynthetic pathway.

## Introduction

Intracellular lipid accumulation, inflammatory responses, and apoptosis are the major pathogenic events of many metabolic disorders, including atherosclerosis and nonalcoholic fatty-liver diseases (NAFLD). Increasing evidence shows that nuclear receptors play critical roles in the regulation of lipid metabolism and inflammatory responses [1], [2]. Nuclear receptors are ligand-activated transcription factors that affect processes as diverse as reproduction, development, inflammation, and general metabolism through regulating target gene expression. Many nuclear receptors function as sensors of cellular cholesterol and lipid levels, and elicit gene-expression changes that maintain lipid homeostasis and protect cells from lipid overload. Examples include receptors for fatty acids (peroxisome proliferator activated receptors, PPARs) [3], [4], oxysterols (liver X receptors, LXRs), bile acids (farnesoid X receptor, FXR), and retinoic acids (retinoic acid receptors, RXRs). In many cases, however, their natural ligands—such as orphan nuclear receptors—remain unknown. To further identify authentic lipid ligands of nuclear orphan receptors becomes more and more important.

Oxysterols play an important role in maintenance of cholesterol homeostasis and lipid metabolism [5], [6]. Oxysterols suppress cholesterol biosynthesis through degradation of mRNA of 3-hydroxy-3-methylgutaryl-CoA reductase (HMGCR) and stimulates cholesterol efflux and clearance via activation of LXR and subsequently increasing gene expression of ATP-binding cassette subfamily A1 (ABCA1) and G5/8 (ABCG5/8) in the liver [7], [8]. On the other hand, LXR activation up-regulates the expression of SREBP-1c, which in turn up-regulates at least 32 genes involved in lipid biosynthesis and transport [9]. Therefore, LXR activation could have a profound effect on serum cholesterol levels, but its inappropriate activation of SREBP-1c could lead to hepatic steatosis and hypertriglyceridemia due to the elevated fatty acid and triglyceride synthesis [10].

Oxysterol sulfation as a regulatory pathway has grown out of a series of studies in the past seven years, including discovery of a novel oxysterol sulfate, identification of a key enzyme hydroxysterol sulfotransferase 2B1b (SULT2B1b) in oxysterol sulfate biosynthesis, and investigation into the role of oxysterol sulfates in regulation of lipid metabolism, inflammatory responses, and cell proliferation [11]. The previous report has shown that bile acid biosynthesis via the acidic, “alternative”, pathway was limited by mitochondrial cholesterol uptake. This barrier could be overcome by increasing expression of the mitochondrial cholesterol transporter StarD1. This suggests a physiological role for StarD1. Increases in StarD1 expression also led to up-regulation of biliary cholesterol secretion and downregulation of cholesterol, fatty acid, and triglyceride biosynthesis [12], [13], [14], [15], and inhibition of inflammation and apoptosis [16], [17]. A search for these regulatory effects' mechanisms led to the discovery of a novel sulfated oxysterol, 5-cholesten-3β, 25-diol, 3-sulfate (25HC3S) with potent regulatory properties [18], [19]. The results imply that StarD1 serves as a sensor for high levels of intracellular cholesterol. StarD1 delivers cholesterol into mitochondria for biosynthesis of regulatory oxysterols, which maintenances lipid homeostasis [18], [11]. 25HC3S can be biosynthesized by sterol sulfotransferase SULT2B1b using 25-hydroxycholesterol (25HC) as the substrate via oxysterol sulfation [20]. Functional studies have shown that this regulatory pathway plays an important role in lipid metabolism, inflammatory responses, and cell proliferation through regulating nuclear-receptor activity [19], [21], [22], [23], [24], [25], [26]. All the established studies indicate that oxysterol sulfation is a novel regulatory pathway in regulation of lipid metabolism, inflammation, and cell proliferation.

Exogenous administration of 25HC3S decreases both SREBP-1c and SREBP-2 expression, blocks the SREBP-1c processing, represses the expression of key enzymes including acetyl-CoA carboxylase-1 (ACC-1), fatty acid synthase (FAS) and HMGR in lipid metabolism, subsequently decreases intracellular neutral lipid and cholesterol levels [19], [21], [22], [27]. The results indicate that 25HC3S appears to act as a LXR antagonist and as a cholesterol satiety signal, suppressing fatty acid and triglyceride biosynthesis via inhibiting LXR/SREBP pathway [21]. Moreover, 25HC3S increases IκBα expression, blocks TNFα-induced IκBα degradation, and decreases nuclear NFκB level. In contrast, 25HC acts in an opposite manner: inducing IκBα degradation and NFκB nuclear translocation [22], [24]. These results indicate that 25HC3S is also involved in inflammatory responses, which may represent a link between inflammatory pathways and the regulation of lipid homeostasis via oxysterol sulfation.

In the present study, we identified another novel sulfated oxysterol, 5-cholesten-3β,25-diol, disulfate (25HCDS). Administration of 25HCDS substantially increased expression of genes encoding PPARγ, PPARγ coactivator 1 alpha (PGC-1α), and IκB, and decreased hepatic triglyceride and cholesterol levels by suppressing LXR-SREBP-1c/SREBP-2 signaling pathway in mouse NAFLD models. These findings provide strong evidence that 25HCDS is another potent regulator involved in lipid metabolism and inflammatory responses.

## Materials and Methods

### Materials

Cell culture reagents and supplies were purchased from GIBCO BRL (Grand Island, NY); 25-hydroxycholesterol from New England Nuclear (Boston, MA). THP-1 and HepG2 cells were obtained from American Type Culture Collection (Rockville, MD). The reagents for real time RT-PCR were from AB Applied Biosystems (Warrington WA1 4 SR, UK). The chemicals used in this research were obtained from Sigma Chemical Co. (St. Louis, MO) or Bio-Rad Laboratories (Hercules, CA). Polyclonal rabbit antibodies against SREBP-1, SREBP-2, and HMG-CoA reductase were purchased from Santa Cruz Biotechnology (Santa Cruz, CA). All solvents were obtained from Fisher (Fair Lawn, NJ) unless otherwise indicated. The enhanced chemiluminescence (ECL) reagents were from Amersham Biosciences (Piscataway, NJ). Oasis®Wax Cartridges were purchased from Waters Corporation (Milford, MA). The testosterone and 27-hydroxycholesterol were obtained from Research Plus Inc. (Bayonne, NJ). LK6 20×20 cm normal phase thin layer chromatography (TLC) plates were purchased from Whatman Inc. (Clifton, NJ).

### Characterization of Novel Cholesterol Metabolite

#### Adenovirus Preparation and Propagation

The adenovirus construct used in this study was obtained through the Massey Cancer Center Shared Resource Facility of the Virginia Commonwealth University as previously described [28].

#### Cell Culture and Nuclear Fraction of Primary Rat Hepatocytes Isolation and Lipids Extraction

Primary rat hepatocyte (PRH) cultures, prepared as previously described [18], were seeded on 150 mm tissue culture dishes (∼2.5×10^7^ cells) in Williams' E medium containing dexamethasone (0.1 µM). Cells were maintained in the absence of thyroid hormone. Twenty-four hours after plating, culture medium was removed, and 4 ml of fresh medium was added. Cells were then infected with recombinant adenovirus encoding either the StarD1 cDNAs followed the human cytomegalovirus (CMV) promoter (Ad-CMV-StarD1) or no cDNA, as a control virus. The viruses were allowed to incubate for 2 hrs in minimal culture medium with gentle shaking of the plates every 15 minutes. After 2 hrs of infection, unbound virus were removed and replaced with 20 ml of fresh medium. After 48 hrs, cells were then harvested and processed for nuclear isolation as described [28]. Briefly, cells were disrupted by Dounce homogenization in buffer A (10 mM HEPES-KOH at pH 7.6, 1.5 mM MgCl_2_, 10 mM KCl, 0.5 mM dithiothreitol, 1 mM sodium EDTA, 1 mM EGTA) and spun at 1,000× g for 10 mins. The nuclear pellet was further fractionated by resuspension in 2.5 ml of a 1∶1 mixture of buffer A and buffer B (2.4 M Sucrose, 15 mM KCl, 2 mM sodium EDTA, 0.15 mM spermine, 0.15 mM spermidine, 0.5 mM dithiothreitol) and centrifuged at 100,000× g for 1 hr at 4°C through a 1 ml cushion of 3∶7 mixture of buffer A and B. The washed nuclear pellet was resuspended in buffer A containing 0.5% (v/v) nonidet P-40 and centrifuged at 1000× g for 10 min at 4°C. The supernatant is designated as purified nuclei.

The purified nuclei were re-suspended and digested by 2 mg/ml of DNase I in 50 mM of acetic buffer, pH 5.0, 10 mM MgCl_2_ at 37°C for 2 hrs. After centrifugation at 10,000× g for 20 mins, the pellets were further digested by 2 mg/ml of proteinase K in phosphate buffered saline solution (PBS) at 50°C for 16 hrs. Total lipids in the digests were extracted by adding 3.3 volumes of chloroform∶methanol (1∶1) and separated into two phases, methanol/water and chloroform phases as previously described [29].

#### HPLC Analysis of Cholesterol Derivatives in Methanol/Water Phase

Total lipid extracts in methanol/water phases were analyzed on an Ultrasphere PTH C-18 column (5 µ×4.6 mm×25 cm; Backman, USA) at 0.8 ml/min flow rate. The column was equilibrated and run in 20 mM KH_2_PO_4_, pH 4.2:acetonitrile∶methanol (1∶3∶6, v/v/v) as the mobile phase. The effluents were monitored at 195 nm and collected every 0.5 min (0.4 ml per fraction) except as indicated. The column was calibrated with tauroursodeoxycholic acid, glycoursodeoxycholic acid, taurocholic acid, glycocholic acid, taurochenodeoxycholic acid, taurodeoxycholic acid, and progesterone.

#### Purification of Sulfated Oxysterols

Sulfated oxysterols were further purified from liver lipid extracts in the methanol/water phases. The extracts were dried under nitrogen stream and dissolved in 50% phosphate buffer saline (PBS) in acetonitrile (loading buffer). The solution was passed through Oasis Wax 6cc Cartridge. The column was washed with 10 volumes of the loading buffer, 10 volumes of water, 10 volumes of methanol, 2 volumes of 10% methanol in water, and 5% NH_4_OH in 10% methanol. The sulfated oxysterols were eluted by 3 ml of 5% NH_4_OH in 80% methanol and analyzed by LC/MS/MS.

#### Reverse Phase Liquid Chromatography/Tandem Mass Spectrometry/Mass Spectrometry (LC/MS/MS) Analysis of Sulfated Oxysterols

Samples to be analyzed were constituted in methanol∶water (20∶80, v/v) and separated on a ThermoKeystone Aquasil C18 column (5 µm, 2.1 mm×100 mm). The mobile phase was consisted of (A), 0.1% formic acid in water, and (B), 0.1% formic acid in acetonitrile. The 20 min gradient was as follows: 0–10.0 min, 10%–95% B linear; 10.0–15.0 min, 95% B; 15.0–15.1 min, 95%-10% B linear; 15.1–20.0 min, 10% B. The elution stream (0.3 ml/min) from the HPLC apparatus was introduced into a MDS Sciex ABI 4000 Triple Quadrapole Mass Spectrometer (MDS Sciex, Toronto, Canada) with a Turbo Ion Spray ionization (ESI) source for the analyses. The mass spectrometer was operated in negative ion modes, and data were acquired using both full scan mode as well as the product ion mode for MS/MS (Core Facility, Virginia Commonwealth University School of Pharmacy).

### Chemical Synthesis of 5-Cholesten-3β, 25-Diol, Disulfate

Synthesis of 5-cholesten-3β,25-diol-disulfate (25HCDS): To a solution of the 25-hydroxycholesterol (80 mg, 0.14 mmol) in anhydrous pyridine (800 µL), sulfur trioxide–trimethylamine complex (133 mg, 0.95 mmol) was added, and the suspension was stirred at 50°C at room temperature overnight. To the reaction mixture, 2 ml of acetonitrile were added and evaporated to dryness under N_2_ stream. The pellets were dissolved in 2 ml of 50% PBS, pH 7.2, in acetonitrile (loading buffer) applied to a 6 cc Oasis cartridge (Waters), which had been primed by methanol (15 mL) and water (15 mL). The cartridge was successively washed with the loading buffer (15 ml), methanol (15 mL), 10% methanol (5 ml), and 5% ammonia hydroxide in 10% methanol (15 ml). The retained 25HCDS was eluted with 5% ammonia hydroxide in 60% methanol (10 ml). After dilution with 10 times volume of acetonitrile, the solvents were evaporated to dryness under N_2_ stream, and the 25HCDS was obtained as powdered form. Yield was about 70 mg (∼70%).

^1^H and ^13^C NMR spectra were obtained on a Varian 500 Inova (AS500) instrument at 499.62 MHz and 125.64 MHz, respectively. Flow injection low-resolution mass (LR-MS) spectra were recorded by a Thermo Scientific TSQ Quantum Ultra MS equipped with electrospray ionization (ESI) probe under negative ion mode. Reversed-phase TLC was carried out on pre-coated RP-18F254S plates using methanol–water–acetic acid mixtures (89∶10∶1, v/v/v) as the developing solvent. The spots were visualized by 50% H_2_SO_4_ with heating at 110°C.

### Functional Studies

#### Cell Culture

Human THP-1 monocytes and HepG2 cells were maintained according to the supplier's protocols. THP-1 monocytes were differentiated to macrophages by adding 100 nM of phorbol 12-myristate 13-acetate (PMA) for 5 days. When cells reached ∼90% confluence, 25HCDS in ethanol (the final concentration of ethanol in media was 0.1%) was added. The cells were harvested at the indicated times for protein, mRNA, and lipid analysis.

For study of HMG CoA reductase expression regulation, HepG2 or THP-1 were cultured in the media as described above in the presence or absence of mevinolin (50 µM) and mevalonate (0.5 µM). After culturing for 48 hrs, oxysterols were added and cultured for another 6 hrs. The cells were harvested for determining mRNA levels.

#### Determination of Cholesterol Biosynthesis by TLC

After incubation of THP-1 derived macrophages or HepG2 cells in media containing different concentrations of 25HCDS as indicated for 6 hrs, cells in 60 mm dishes were given 3 ml of the same fresh medium containing 5 µCi of [1-^14^C] acetate. After 2 hr incubation at 37°C, the media was removed. The cells were washed twice with phosphate-buffered saline (PBS), harvested with rubber police as described, and collected in microcentrifuge tubes. The cells were sedimented by centrifugation, and the pellets were washed three times by resuspension and sedimentation. The cellular pellets were resuspended in 0.3 ml of PBS. The total lipids were extracted and separated by adding 3 volumes of chloroform∶methanol (1∶1) as previously described [18]. [^14^C] Cholesterol and hydroxycholesterols were isolated into chloroform phase and separated on TLC (toluene∶acetyl acetate, 2/3, v/v/). [1-^14^C] acetate derivatives were visualized by Image Reader, Fujifilm BAS-1800 II as previously described [19].

[1-^14^C] Acetate derivatives in the chloroform phase were analyzed by HPLC on an Ultrasphere Silica column (5 µm×4.6 mm×25 cm; Backman, USA) using HP Series 1100 solvent delivery system (Hewlett Packard) at 1.3 ml/min flow rate. The column was equilibrated and run in a solvent system of hexane∶isopropanol∶glacial acetic acid (965∶25∶10, v/v/v), as the mobile phase. The effluents were collected every 0.5 min (0.65 ml per fraction) except as indicated. The counts in [^14^C] acetate derivatives were determined by Scintillation Counting. The column was calibrated with [^14^C] cholesterol, [^3^H]25-hydroxycholesterol, and [^14^C] 27-hydroxycholesterol.

#### Determination of mRNA Levels by Real-Time RT-PCR

Total RNA was isolated with SV Total RNA Isolation Kit (Promega, Madison, WI), which included DNase treatment. Total RNA, 2 µg, was used for the first-strand cDNA synthesis as recommended by the manufacturer (Invitrogen, Carlsbad, CA). Real-time RT-PCR was performed using SYBR Green as indicator on ABI 7500 Fast Real-Time PCR System (Applied Biosystems, Foster City, CA). Amplifications of β-actin and GAPDH were used as internal controls. Relative messenger RNA (mRNA) expression was quantified with the comparative cycle threshold (Ct) method and was expressed as 2^−ΔΔCt^ as describe before [30].

#### In vivo Study using Fatty Liver Animal Model

Animal studies were approved by Institutional Animal Care and Use Committee of McGuire Veterans Affairs Medical Center and were conducted in accordance with the Declaration of Helsinki, the Guide for the Care and Use of Laboratory Animals, and all applicable regulations. To examine the effect of 25HCDS on diet-induced lipid accumulation in sera and liver, 8-week-old female C57BL/6J mice (Charles River, Wilmington, MA) were fed high fat diet (HFD) (Harlan Teklad, Madison, WI) containing 42% kcal from fat, 43% kcal from carbohydrate, 15% kcal from protein and 0.2% cholesterol for 16 weeks and changed to normal diet three days before the end period as previously reported [23]. All mice were housed under identical conditions in an aseptic facility and given free access to water and food. After 10-week's HFD feeding the mice were intraperitoneally injected with vehicle solution (ethanol/PBS) or 25HCDS (25 mg/kg) twice a week for 6 weeks. At the end of the experiment, all mice were fasted overnight before blood and liver samples were collected. Triglyceride, total cholesterol, high density lipoprotein-cholesterol, glucose, alkaline phosphatase (ALK), alanine aminotransferase (ALT), and aspartate aminotransferase (AST) in sera were measured using standard enzymatic techniques in the clinical laboratory at McGuire Veterans Affairs Medical Center.

#### Quantification of hepatic lipids

Liver tissues were homogenized, and lipids were extracted with a mixture of chloroform and methanol (2∶1) and filtered. The extracts, 0.2 ml, were evaporated to dryness and dissolved in 100 µl of isopropanol containing 10% of triton X-100 for cholesterol assay (Wako Chemicals USA, Richmond, VA), NEFA solution (0.5 g of EDTA-Na_2_, 2 g of Triton X-100, 0.76 ml of 1N NaOH, and 0.5 g of sodium azide/l, pH 6.5) for free fatty acid assay (Wako Chemicals USA, Richmond, VA), or isopropanol only for triglyceride assay (Fisher Scientific, Pittsburgh, PA), respectively. All of the assays were performed according to the manufacturer's instructions. Each lipid concentration was normalized to total protein of liver.

#### Statistics

Data are reported as the mean ± standard deviation. Where indicated, data were subjected to t-test analysis and determined to be significantly different if p<0.05.

## Results

**Figure 1:**
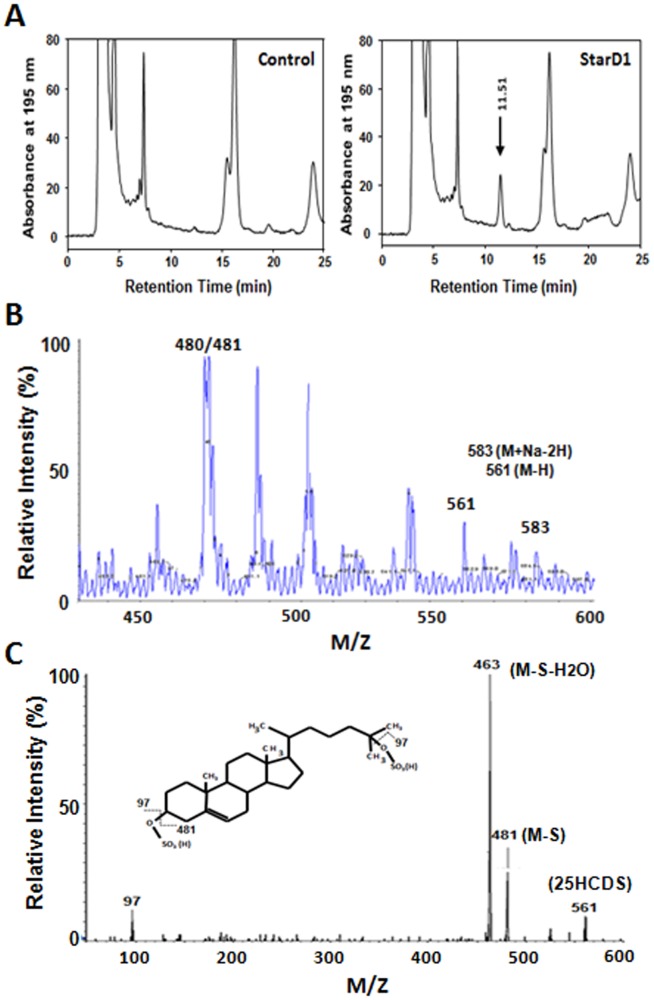
Characterization of nuclear oxysterol as 5-cholesten-3β, 25-diol disulfate by negative ion-triple quadruple mass spectrometry. Forty-eight hours following the indicated recombinant adenovirus infection, cells were harvested. Nuclear fraction was isolated and extracted by the Folch method. The lipids in the methanol/water phase were analyzed by HPLC by monitoring at 195 nm (**Panel A**). In each case, the nuclear methanol/water extracts of the equivalent of 5×10^6^ cells were loaded. The elution profiles represent one of the five sets of experiments (the profiles are highly repeatable). HPLC/MS negative full scan spectrum, HPLC-MS elution profile sorted with mass ion 80, of extract from primary rat hepatocyte is shown in **Panel B**. The MS-MS product spectrum of the mass 561 and its fragmentation diagrams are shown in **Panel C**. The product spectrum of mass ion 583 is similar to that of 561 (data not shown). Each fragment represents negative ion of the chemical structure fragments m/z 561 and m/z 583.

Analysis of the lipid composition in the methanol/water phases extracted from nuclear fractions following overexpression of StarD1 protein showed a unique peak with retention times at 11.5 min in HPLC elution profile (**Fig. 1A**). The results are consistent with previous reports, and the peak was believed to be cholesterol metabolites [18]. To further characterize the chemical structure of the peak, the HPLC purified cholesterol metabolites were analyzed by liquid chromatography-tandem mass spectrometry (LC/MS/MS). The LC/MS Q1 full scan spectrum sorted with m/z 80 showed two bigger molecule ions, m/z 561 and 583 and two major molecular ions 480 and 481 (**Fig. 1B**). The latter two ions have been characterized as 25HC3S [18]. These two molecular ions were further analyzed by HPLC/MS-MS under negative ionization mode. The product scan of m/z 561 has been shown in **Fig. 1C**. The characteristic fragment ions of these two bigger molecules are very similar (data not shown). Fragment ions were observed at m/z = 97, 463, and 481 in the product scan spectrum of m/z 561 (**Fig. 1C**). These observed fragment ions indicate that the nuclear oxysterol is a disulfated oxysterol with a sulfate group on 3-OH and 25 position (18) and a hydroxyl group on side chain, m/z 59, (molecular mass 562 = 160 (disulfate)+16 (O)+386 (cholesterol). Referenced with the data from previous reports [18], the nuclear oxysterol derivative was assumed as 5-cholesten-3β, 25-diol disulphate (25-hydroxycholesterol 3,25-disulfate, 25HCDS) (**Fig. 1C**). This structure has been further confirmed by chemically synthesized 25HCDS as stated below.

### Chemical Synthesis of the Nuclear Oxysterol, 5-cholesten-3β, 25-diol, Disulfate

**Figure 2:**
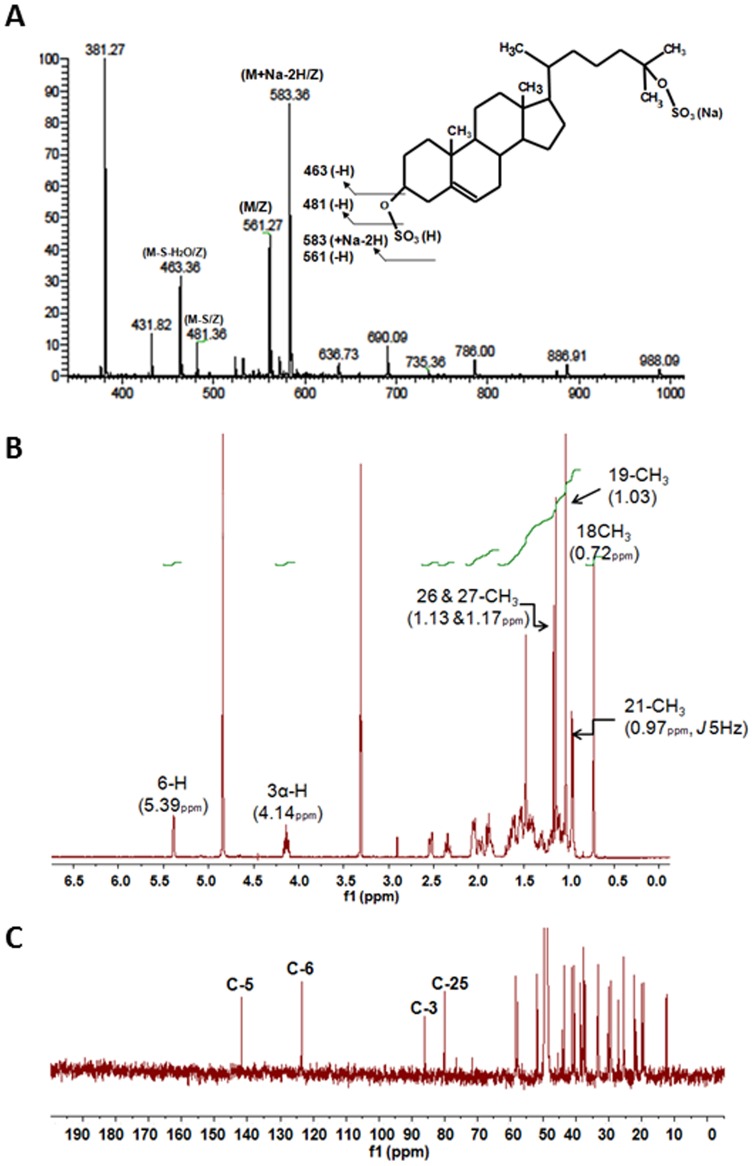
Analysis of chemically synthesized 25HCDS: MS spectrum and ^1^H NMR spectrum of 25HCDS. (**A**) Negative ion flow injection mass spectrum of 25HCDS. m/z, 583(M+Na-2H), 561 (M-H) 481 (M-S) and 463 (M-S-S-H2O) were observed as major ions. This fragmentation pattern is similar to that obtained from the nuclear compound (Fig. 1C); (**B**) 1H NMR spectrum of 25HCDS; the sulfated 3α-H resonated at 4.14 ppm which was ca 0.7ppm downfield compared to that of unsulfated 3α-H in 25HC. All other chemical shifts and their spin couplings observed were similar to those of 25HC and 25HC3S; and (**C**) 13C NMR spectrum of 25HCDS; the C-3 and C-25 resonated at 86 and 80 ppm, which were δ 14 ppm and δ 9 ppm downfield, respectively, indicating that both positions are sulfated.

To confirm its structure and study its role in cellular lipid homeostasis and inflammatory responses, 25HCDS was chemically synthesized and purified. MS analysis of the synthesized compound shows the same molecular mass ion, m/z 561 and m/z 583 (+Na) as the authentic nuclear oxysterol, and the purified product was not contaminated by the starting material, 25-hydroxycholesterol, m/z 401. LR-MS (ESI-negative), *m*/*z*: 583.4 (M+Na-2H, 88%), 561.3 (M-H, 46%), 481.4 (M-SO_3_-H, 11%), 463.4 (M-H_2_SO_4_-H, 34%), 431.82 (14%), 381.27 (100%) (**Fig. 2A**). ^1^H NMR (CD_3_OD) δ: 0.72 (3H, s, 18-CH_3_), 0.97 (3H, d, *J* 5.0 Hz, 21-CH_3_), 1.03 (3H, s, 19-CH_3_), 1.14 (6H, s, 26- and 27-CH_3_), 4.14 (1H, br. m, 3α-H), 5.39 (1H, br. s, 6-H) (**Fig. 2B**). ^13^C NMR (CD_3_OD) δ: 12.45, 19.37, 19.90, 21.82, 22.29, 25.45, 25.51, 27.05, 27.12, 29.39, 29.44, 30.13, 33.16, 33.37, 37.26, 37.32, 37.50, 38.60, 40.52, 41.27, 43.65, 51.78, 57.71, 58.37, 79.98, 85.93, 123.44, 141.71 (**Fig. 2C**). The results indicate that the synthesized molecule is 5-cholesten-3β, 25-diol, disulphate (25HCDS) and matches the molecule found in the hepatocyte nuclear fraction.

### 25HCDS Inhibits Lipid Biosynthesis at Transcriptional Levels

**Figure 3:**
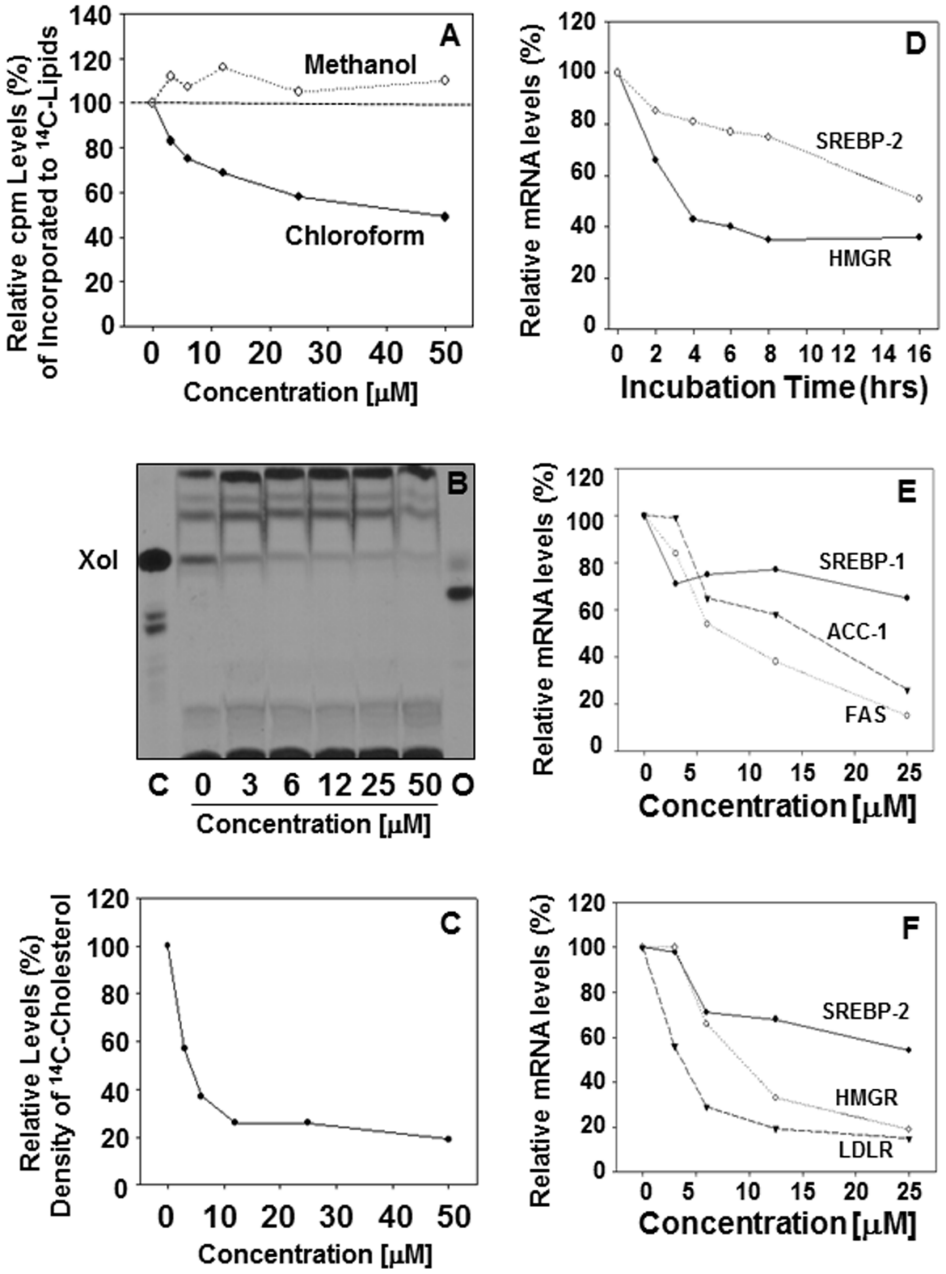
Effects of 25HCDS on lipid metabolism in HepG2 cells. After incubation of the 25HCDS-treated HepG2 cells with [1-^14^C] acetate for 2 hrs, the total neutral lipids were extracted and Folched. [^14^C]-Counts (neutral lipids) in methanol and chloroform phases are shown in **Panel A**. TLC analysis of the [^14^C]-acetate derivatives extracted from the equivalent of 5×10^6^ cells per each lane is shown in **Panel B**, where the Xol represents cholesterol; the [^14^C]-acetate derivative cholesterol was further relatively quantitated by HPLC. The column was calibrated by standards [^14^C]cholesterol, [^14^C]27-hydroxycholesterol, and [^3^H]25-hydroxycholesterol. HPLC analysis of the [^14^C]-acetate derivatived cholesterol is shown in **Panel C**. Real time RT-PCR analysis of SREBP-2 and HMG CoA reductase in HepG2 cells treated with 25HCDS at indicated time is shown in **Panel D**; SREBP-1c, ACC, and FAS mRNA levels, at indicated concentration, are shown in **Panel E**; SREBP-2, HMG-CoA reductase, and LDLR are shown in **Panel F**. The expression levels were normalized toGAPDH. Each value represents mean of three measurements.

To examine the effects of 25HCDS on cholesterol biosynthesis, the rates of cholesterol synthesis were determined. The effects of 25HCDS on lipid biosynthesis were summarized in Fig. 3. After addition of 25HCDS, the cells were cultured for 6 hrs and with [1-^14^C] acetate for an additional 2 hrs. Total lipids were extracted and partitioned. The ^14^C-lipids in the chloroform phase as decreased significantly in a concentration-dependent manner and increased slightly in the methanol phase as shown in **Fig. 3A**. The results indicate that neutral lipid biosynthesis has been suppressed. TLC analysis of lipid in chloroform phase showed that [^14^C] cholesterol and [^14^C] cholesterol ester, were synthesized but [^14^C] oxysterol was not detected (**Fig. 3B**). Free [^14^C] cholesterol was found significantly decreased following addition of 25HCDS (**Fig. 3B**). The decrease was concentration dependent (**Fig. 3B**). The decreased amounts of [^14^C]cholesterol bands on the TLC were confirmed by HPLC analysis (**Fig. 3C**). The effect of 25HCDS on suppression of cholesterol biosynthesis is slightly stronger than that of 25HC3S [19].

To investigate how 25HCDS inhibits lipid biosynthesis, total RNA was isolated from the 25HCDS-treated HepG2 cells. The mRNA levels of ACC and FAS for triglyceride synthesis, and HMGCR for cholesterol synthesis were determined by real time RT-PCR. As shown in **Fig. 3D**, 25HCDS decreased expression of SREBP-2 and HMGCR in HepG2 **in time dependent manner**. As shown in **Fig. 3E and F**, 25HCDS decreases expression of SREBP-1c, and its response genes, ACC and FAS (**3E**), and SREBP-2, HMGCR, and LDLR mRNA levels (**3F**) in concentration dependent manner. As indicated in other studies, ACC-1, FAS are the target genes of SREBP-1 pathway, and HMGR and LDLR are the target genes of SREBP-2. The trend in expression of target genes was consistent with the decreases in expression of SREBP1/2 (**Fig. 3E and F**). Similar results have been observed in THP-1 macrophages and primary rat hepatocytes (data not shown). These results indicate that 25HCDS decreases expression of key genes involved in lipid biosynthesis via SREBP pathway.

### Effects of 25HCDS on Lipid Homeostasis and Inflammation in HFD-fed Mice

**Figure 4:**
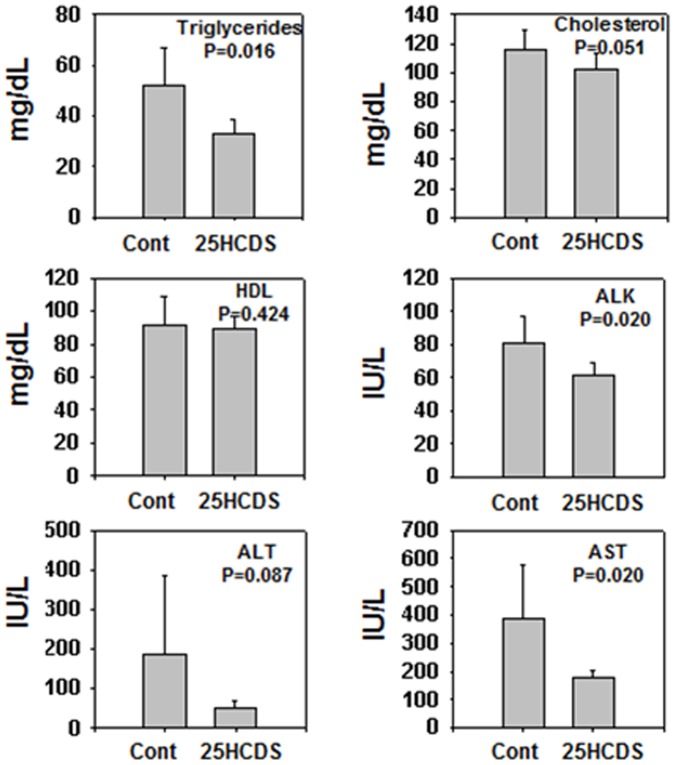
25HCDS decreases serum lipid levels and improves liver function in mouse NAFLD models. Eight-week-old C57BL/6J female mice treated with either 25HCDS or vehicle every three days for six weeks and fasted overnight. Plasma triglyceride, total cholesterol, and high-density lipoprotein (HDL), alkaline phosphatase (ALK), alanine aminotransferase (ALT), and aspartate aminotransferase (AST) were determined. All the data are expressed as mean ± SD with p value when 25HCDS treated versus vehicle-treated mice liver.

To study the effects of long-term treatment of 25HCDS on lipid homeostasis, 10-week-old C57BL/6J female mice were fed with a HFD for 10 weeks, and then, randomly divided into two groups: 25HCDS or vehicle treatment respectively by peritoneal injection twice a week for six weeks. During the treatment, the mice were fed with HFD, and body weights and caloric intake were monitored (no significant difference, data not shown). To decrease variation of serum lipid profile, after 6 weeks' treatment, the high fat diet was changed to normal chew diet three days before the mice were sacrificed after overnight fasting. The liver weight had no significant difference (data not shown). Serum lipid analysis showed that the administration of 25HCDS significantly decreased triglyceride and cholesterol levels by 15% and 20% respectively. ALK, ALT and AST activities were also greatly decreased by 25HCDS treatment (**Fig. 4**).

**Figure 5:**
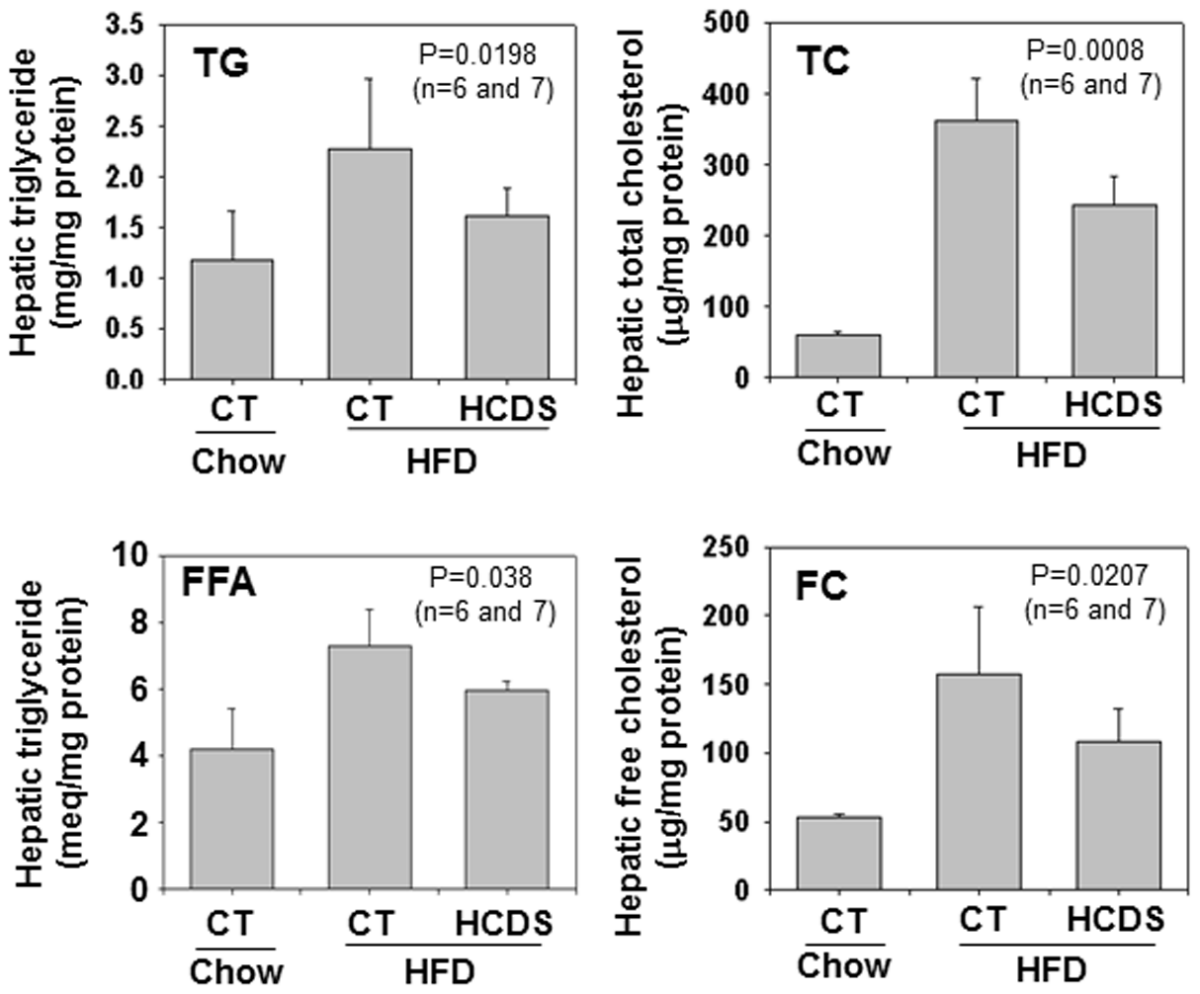
25HCDS decreases lipid accumulation in the liver tissue in mouse NAFLD models. Animals were peritoneal-injected with 25HCDS once every 3 days for 6 weeks. Hepatic triglyceride, free fatty acid, total cholesterol, free cholesterol, cholesterol ester, free fatty acid, and triglyceride were determined as described in Methods. Each individual level was normalized by protein concentration. All the data are expressed as mean ± SD with p value when 25HCDS treated versus vehicle-treated mice liver.

To study the effect of 25HCDS on hepatic lipid metabolism, hepatic lipid levels and related gene expression were determined. As previously reported, HFD-fed increased triglyceride, total cholesterol, free fatty acid, and triglyceride levels in the liver compared to chow-fed mice (**Fig. 5**). These increases were significantly reduced by 25HCDS administration by 25%, 20% and 18% (p<0.05), respectively, as shown in **Fig. 5**.

Gene expression study showed that 25HCDS administration significantly decreased the expression of key enzymes and receptors involved in biosynthesis of free fatty acid, triglyceride, and cholesterol (**Table 1**). Dysregulation of lipid metabolism is frequently associated with inflammatory conditions. 25HCDS treatment significantly suppressed the expression of TNFα and IL1β, by 50% and 36%, respectively (**Table 2**). These results are consistent with the liver functional assay where it shows that 25HCDS suppresses liver inflammatory responses and improves liver regeneration as decreasing alkaline phosphotase (ALK) activity in sera. Interestingly, 25HCDS increased expression of PGC-1α by 2-fold in the liver. As a result, 25HCDS may regulate lipid metabolism and inflammatory responses via LXR, PPARγ and PGC-1αsignaling.

**Table 1:** Relative Hepatic mRNA Expression Involved in Lipid Metabolism in Mice Fed on a HFD with or without 25HCDS.

Gene Name	Gene description	HFD (n = 6)	HFD+25HCDS (n = 7)
**Fatty acid biosynthesis**			
SREBP-1c	Sterol regulatory element-binding protein-1c	1.0±0.36	**0.64±0.14** *
ACC1	Acetyl-CoA carboxylase 1	1.0±0.31	0.86±0.18
FAS	Fatty acid synthase	1.0±0.27	**0.68±0.17** *
**Triglyceride metabolism**			
GPAM	Glycerol-3-phosphate acyltransferase	1.0±0.10	**0.74±0.18** *
MTTP	Microsomal triglyceride transfer protein	1.0±0.11	0.94±0.17
PLTP	Phospholipid transfer protein	1.0±0.32	**0.68±0.21** *
**Cholesterol Metabolism**			
SREBP-2	Sterol regulatory element-binding protein-2	1.0±0.18	1.12±0.25
HMGR	Hydroxy-methylglutaryl-coenzyme A reductase	1.0±0.16	**0.84±0.07**
LDLR	Low density lipoprotein receptor	1.0±0.43	**0.62±0.08** *
CD36 (FAT)	Fatty acid translocase	1.0±0.52	0.69±0.29

Animals were treated as described in Methods.

All values are expressed as the mean ± SD; n = 6–7.

* p<0.05 compared with HFD mice.

Abbreviations: HFD, high fat diet.

**Table 2:** Relative Hepatic mRNA Expression Involved in Inflammatory Responsesin in Mice Fed on a HFD with or without 25HCDS.

Gene Name	Gene description	HFD (n = 6)	HFD+25HCDS (n = 7)
PGC-1α	Peroxisome proliferator-activated receptor gamma coactivator-1α	1.0±0.27	**2.11±0.82** *
PPARγ	Peroxisome proliferator-activated receptor gamma	1.0±0.42	1.27±0.52
IκBα	Nuclear factor of kappa light polypeptide gene enhancer in B-cells inhibitor α	1.0±0.25	**1.35±0.27** *
TNFα	Tumor necrosis factor α	1.0±0.28	**0.50±0.21** **
IL1α	Interleukin 1α	1.0±0.35	1.02±0.20
IL1β	Interleukin 1β	1.0±0.21	**0.64±0.16** **

Animals were treated as described in Methods.

All values are expressed as the mean ± SD;

* p<0.05,

** p<0.01 compared with HFD mice.

Abbreviations: HFD, high fat diet.

## Discussion

**Figure 6:**
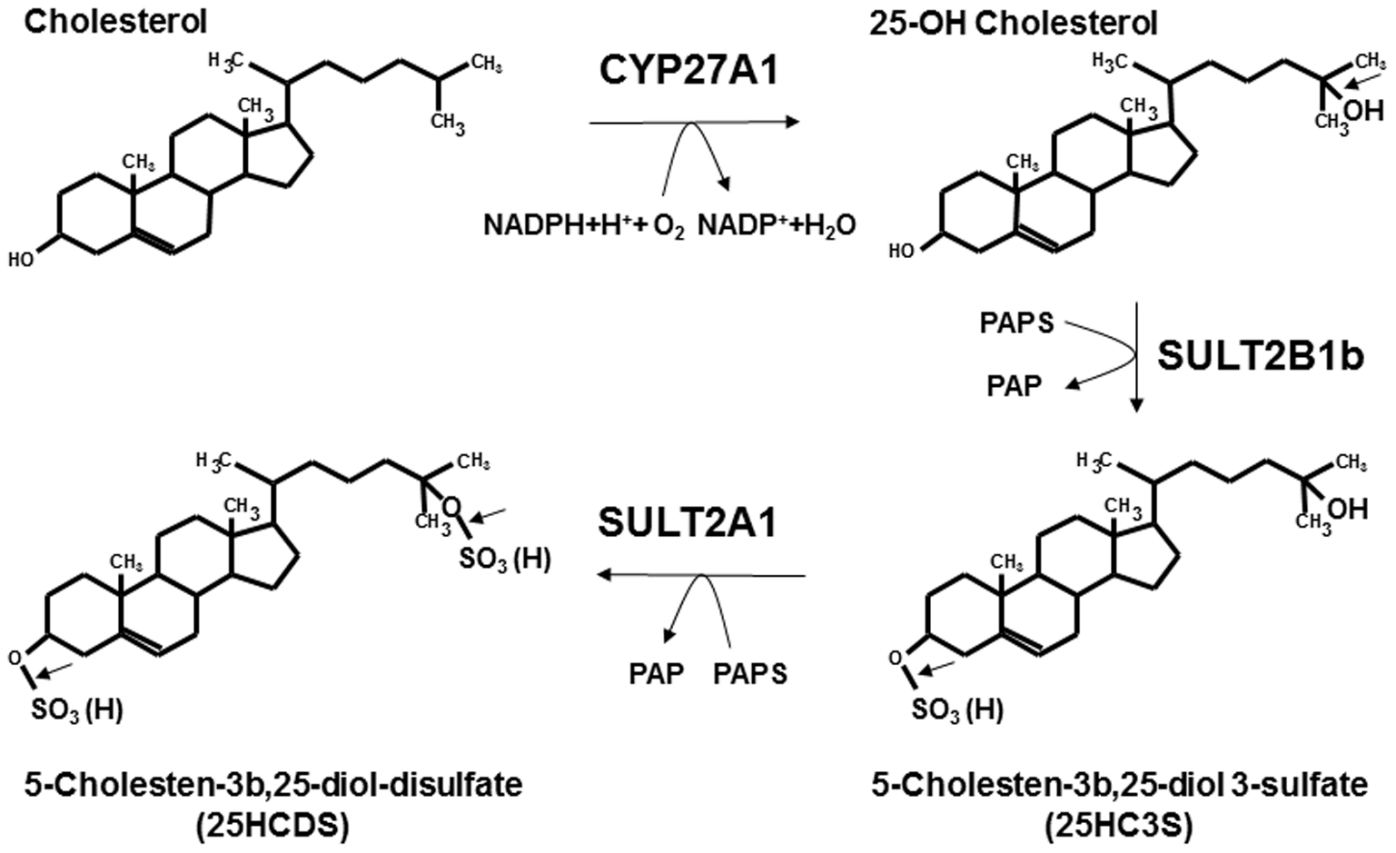
Biosynthetic pathway of 25HCDS. Cholesterol is hydroxylated by CYP27A1 in the mitochondria to 25HC, which is subsequently sulfated at the 3β-position by SULT2B1b to form 25HC3S and further sulfated at the 25-hydroxyl group by SULT2A1 in cytosol to synthesize 25HCDS.

The function of oxysterol sulfation has extensively been investigated in our laboratories [11]. The first discovered oxysterol sulfate, 25HC3S, functions in vitro and in vivo as a regulatory molecule: decreasing cholesterol and triglyceride biosynthesis, suppressing inflammatory responses, and promoting hepatocyte proliferation [21], [22], [23], [24], [25], [26], [27], [31]. 25HC3S is biosynthesized by CYP27A1 and SULT2B1b [20]. The present work has shown that 25HC3S can be further sulfated to be another novel cholesterol metabolite, 25HCDS. It has been reported that SULT2A1 is able to sulfate hydroxyl group(s) on the side-chain in oxysterol molecules with much less specificity than SULT2B1b and be able to synthesize oxysterol disulfates [32]. It is reasonable to propose that 5-cholesten-3β, 25-diol disulfate (25HCDS) is biosynthesized from cholesterol by CYP27A1 to form 25-hydroxycholesterol (25HC), sulfated by SULT2B1b to form 25-hydroxycholesterol 3-sulfate (25HC3S), followed by further-sulfated by SULT2A1 to generate 25-hydroxycholesterol di-sulfate as illustrated in **Fig. 6**. Further experiments are needed to confirm the pathway.

The process of 25HCDS biosynthesis, oxysterol sulfation, may represent a novel regulatory pathway, which mediates nuclear receptor activities in hepatocytes. When intracellular cholesterol levels are increased, mitochondrial cholesterol transport protein, StarD1, delivers cholesterol into mitochondria, where regulatory oxysterols, such as 25HC and 27HC, are synthesized by CYP27A1 [20], [33]. These oxysterols in turn activate LXR, and subsequently up-regulate expression of its target genes involved in cholesterol, fatty acid, and triglyceride biosynthesis [6], [34], [35], [36]. In addition, 25HC activates LXR, down regulates newly synthesized cholesterol biosynthesis by inhibiting HMGR expression and increases ABCA1 mediated cholesterol secretion from the cells (HDL formation). Oxysterols can be further sulfated to form oxysterol sulfates, such as 25HC3S and 25HCDS, which inactivate LXRs, suppress SREBP-1c processing, indicating that these sulfated oxysterols decrease intracellular lipid levels by inhibiting their biosynthesis. It is well known that the alternative pathway of bile acid biosynthesis involves the transporting of cholesterol into the mitochondria where the cholesterol is hydroxylated and then converted to bile acids. However, the recent reports that oxysterols and oxysterol sulfates generated by this pathway play an important role in lipid metabolism, inflammatory responses, and cell proliferation, indicating that this pathway is far more than only cholesterol degradation or bile acid biosynthesis [11]. It has been reported that 7-ketocholesterol can be sulfated to be 7-ketocholesterol 3 sulfate [37]; 24-hydroxycholesterol, 24-hydroxycholesterol 3-sulfate [32]; 24,25-epoxycholesterol, 24,25-epoxycholesterol 3-sulfate; cholesterol, cholesterol sulfate. Cholesterol sulfate has been reported to inhibit gluconeogenesis [40] but 25HCDS does not (data not shown). Furthermore, the effects of oxysterols or sterols are completely different to those of the sulfated ones, indicating that intracellular oxysterol or sterol sulfation represents a novel regulatory mechanism involved in many biological events.The liver plays a pivotal role in the maintenance of lipid homeostasis. Accumulation of lipids in liver tissues leads to nonalcoholic fatty liver diseases (NAFLD). The spectrum of NAFLD ranges from simple non-progressive steatosis to progressive nonalcoholic steatohepatitis (NASH) that results in liver cirrhosis and hepatocellular carcinoma [38]. The accumulation of triglycerides and associated lipids and the occurrence of liver inflammation in the hepatocytes are believed to be the major pathogenic factors for the development of the diseases [39]. Lowering lipid levels is an important element of successful NAFLD therapy. However, there is no approved treatment for NAFLD currently. The discovery of the novel cholesterol metabolites, 25HC3S and 25HCDS, which decrease intracellular lipid levels, has laid the groundwork for the development of the better therapies.

The present study shows for the first time that the cholesterol metabolite, 25HCDS, inhibits SREBP-1c/2 expression and activity in vitro and in vivo; increases PPARγ and PGC-1α expression. It is well-documented that SREBPs control lipid biosynthesis, PPARγ regulates inflammatory responses, and PGC-1α controls energy homeostasis. Thus, the results provide evidence that 25HCDS is a potent regulator, which plays an important role in maintenance of hepatic lipid homeostasis and inflammatory responses.
